# Reproducing the Hemoglobin Saturation Profile, a Marker of the Blood Oxygenation Level Dependent (BOLD) *f*MRI Effect, at the Microscopic Level

**DOI:** 10.1371/journal.pone.0149935

**Published:** 2016-03-03

**Authors:** Constantinos Hadjistassou, Keri Moyle, Yiannis Ventikos

**Affiliations:** 1 Fluidics and Biocomplexity Group, Department of Engineering Science, University of Oxford, Oxford, Parks Road, Oxford, United Kingdom; 2 Faculty of Engineering, University of Auckland, Auckland, New Zealand; 3 Department of Mechanical Engineering, University College London (UCL), Torrington Place, London, United Kingdom; James Cook University, AUSTRALIA

## Abstract

The advent of *functional* MRI in the mid-1990s has catalyzed progress pertaining to scientific discoveries in neuroscience. With the prospect of elucidating the physiological aspect of the Blood Oxygenation Level Dependent (BOLD) effect we present a computational capillary-tissue system capable of mapping venous hemoglobin saturation— a marker of the BOLD hemodynamic response. Free and facilitated diffusion and convection for hemoglobin and oxygen are considered in the radial and axial directions. Hemoglobin reaction kinetics are governed by the oxyhemoglobin dissociation curve. Brain activation, mimicked by dynamic transitions in cerebral blood velocity (CBv) and oxidative metabolism (CMR_O_2__), is simulated by normalized changes in *m* = (ΔCBv/CBv)/(ΔCMR_O_2__/CMR_O_2__) of values 2, 3 and 4. Venous hemoglobin saturation profiles and peak oxygenation results, for *m* = 2, based upon a 50% and a 25% increase in CBv and CMR_O_2__, respectively, lie within physiological limits exhibiting excellent correlation with the BOLD signal, for short-duration stimuli. Our analysis suggests basal CBv and CMR_O_2__ values of 0.6 mm/s and 200 *μ*mol/100g/min. Coupled CBv and CMR_O_2__ responses, for *m* = 3 and *m* = 4, overestimate peak hemoglobin saturation, confirming the system’s responsiveness to changes in hematocrit, CBv and CMR_O_2__. Finally, factoring in neurovascular effects, we show that no initial dip will be observed unless there is a time delay in the onset of increased CBv relative to CMR_O_2__.

## Introduction

Advancing the prevailing understanding between functional neuroimaging and neuronal activity promises to bridge the knowledge gap between cellular neuroscience and brain imaging. With applications ranging from pre-surgical planning to neuroeconomics to unlocking human cognition, a comprehensive interpretation of the Blood Oxygenation Level Dependent (BOLD) effect, upon which *functional* MRI (*f*MRI) is based, holds great potential in the quest of unraveling the processes at the core of brain function.

Even though the human brain can store comparatively small glycogen and oxygen reserves [1], relative to the overall cerebral metabolic turnover, the MR scanner registers a higher degree of hemoglobin oxygenation during functional activation than during rest. Although the underlying physiological causes remain perplexing, experimental and theoretical evidence indicates that the brain (regionally) delivers more oxygen than physiologically needed [2, 3]. The MR signal emanates from the diamagnetic properties of oxyhemoglobin (HbO_2_), which when present at a high enough concentration in a voxel registers an augmentation in the MR signal. It follows that, a higher concentration of oxyhemoglobin attenuates spin dephasing within the NMR-sensitive water molecules as they diffuse through the magnetic field gradients. In turn, this prolongs the transverse relaxation time constant ${\text{T}}_{2}^{*}$, in the vicinity of capillaries and venous blood vessels, such that ${\text{T}}_{2}^{*}$-sensitive pulse sequences are able to discern a measurable increase in the MR signal intensity, ranging from 0.5% to 5%— depending on the strength of the static magnetic field (${\text{B}}_{0}^{{}^{\prime }}$) [4].

Physiologically, the BOLD response is deemed to be connected to changes in several parameters; the most prominent being cerebral blood volume (CBV), cerebral blood velocity (CBv), cerebral metabolic rate of oxygen utilization (CMR_O_2__) and cerebral blood flow (CBF) [5]. Under certain conditions, spanning different spatial and temporal scales, responses between CBF and coupled CBF & CMR_O_2__, and the interactions between these factors (and their causal effects) could foster unfavorable conditions for the manifestation of the BOLD response. Presumably these parameters emphasize the BOLD phenomenon’s sensitivity to subtle physiological changes while rendering the interpretation of the MRI signal more challenging. Most of the proposed quantitative descriptions aimed at explaining the BOLD contrast, including Buxton and Frank [2], Buxton *et al*. [6], Buxton *et al*. [7], Hyder *et al*. [8], Friston *et al*. [9], Friston *et al*. [10], Zheng *et al*. [11], are compartmental models. Compartmental models, predominantly based on *integral equations*, generate useful information about the gross behavior of particular physical quantities of biological systems. Meanwhile, spatially distributed models, such the one presented herein, utilize *differential analysis* which captures the physics of various processes and generates a detailed description at every point of the flow in the capillary and tissue computational domains. The BOLD effect being a transport phenomenon, further or complimentary insight may be gleaned through a spatially discerning approach, with sufficient scope to account for the salient physical mechanisms at play.

The physics of the BOLD contrast, tied to the mechanisms of convection, diffusion and hemoglobin reaction kinetics, for our purposes, will be examined at the microscopic (capillary) scale, almost three orders of magnitude smaller than the volume of a typical functional voxel. At this scale, most of the oxygen is reversibly bound to hemoglobin. Its release emanates from the oxygen cascade, triggered by a drop in tissue partial oxygen pressure (pO_2_). In turn, oxygen exchange is invoked by an increase in CMR_O_2__. Intravascular and parenchymal (tissue) oxygen transport is governed by free diffusion, facilitated diffusion, and convection. Diffusion is a metabolically free transport mechanism whose efficiency diminishes drastically over distances in excess of tens of micrometers [12]. In other circumstances, such as pulmonary oxygen transport, the influence the erythrocyte membrane exerts on diffusive transport could also be considered [13]. Analogous to the role of myoglobin in muscle tissue, oxygen transport in the brain, is believed to be amplified by the combination of the gas with neuroglobin carrier molecules [14], in a process known as facilitated diffusion. In contrast, convection, through the bulk movement of blood itself, is capable of transporting oxygen over longer distances— of order meters in length, at the expenditure of energy (cardiac pumping).

Although the coupling relationship of CBF and CMR_O_2__ has garnered considerable attention in several brain studies [2, 6, 15, 16] from the microscopic (capillary) modeling point of view, cerebral blood velocity (CBv) rather than overall volume flowrate can also be a parameter of interest, with the connection between the two easily derived at the capillary level. Investigating oxygen transport at the microscopic level and its interaction with the transient interplay between CBv, CBF, CBV, and CMR_O_2__ are key to understanding the BOLD contrast. Computational modeling offers a testbed for examining hypotheses, allows control over parameters otherwise difficult to test experimentally and complements the traditional branches of science, namely, theory and experiments.

The present study utilizes a computational “capillary-tissue” system, capable of quantifying the influence of neural activation on the venous capillary oxy- to deoxyhemoglobin saturation. Corrosion casts reveal that certain parts of the human cerebral cortex possess capillaries with a significant straight section [17, 18]. In line with this observation, the capillary-tissue model presented herein was assumed to resemble similar geometry which further simplifies things. Free and facilitated diffusion and convection for hemoglobin and oxygen are considered in both the radial and axial directions. Hemoglobin saturation, a biophysical correlate to the BOLD signal, and the effects of dynamic transitions in CBv and CMR_O_2__, are modeled at the single capillary level.

As we shall show in the “Results” section, a 50% increase in CBv and a 25% elevation in CMR_O_2__, or an *m* = (ΔCBv/CBv)/(ΔCMR_O_2__/CMR_O_2__) = 2, produced a hemoglobin saturation (sO_2_) profile in excellent physiological agreement with recorded signals for short-duration stimuli. Considering neurovascular effects, in the form of a 0–2 s time lag between CMR_O_2__ and CBv, a time delay of 0.1 s generated an sO_2_ response in excellent physiological and temporal agreement with the BOLD signal [19]. Furthermore, no initial dip should be anticipated in the absence of a time lag between activation and hemodynamic response (increased inlet CBv).

## Methods

The developed computational framework presented herein allows for the simulation of blood flow and species transport and reaction kinetics within a capillary complemented by oxygen transport and consumption in the peripheral neuronal tissue. The governing equations of mass, momentum and concentration transport and chemical reaction are solved in a time-accurate three-dimensional framework. Taking advantage of the cylindrical geometry of a capillary, we reconcile the spatial dimensionality of the system by casting the transport equations for an axisymmetric body; this way the entire 3D effect is captured, at a reduced computational cost. Axial and radial diffusion, a cell-depleted, plasma-only region near the capillary wall, detailed velocity evaluation of the convective features of the system, hemoglobin-oxygen reaction characteristics and cortical oxygen consumption are some of the characteristics of the computer model.

Extensive literature search revealed substantial variability in capillary blood velocity which ranged between 0.3 mm/s to 1.2 mm/s [30–32]. Based on a systematic investigation, which explored a wide range of blood velocities and oxygen metabolic rates, we were able to narrow down the basal CBv and CMR_O_2__ to the representative values of 0.6 mm/s and 200 *μ*mol/100g/min (*μ*moles of O_2_ per 100g of tissue per minute), respectively. Owing to the small blood volume retained by the capillary bed, relative to the venal compartment [38], the unclarified degree of capillaries’ distensibility and the sensitivity of the BOLD signal to the venous blood (volume), the formulated model does not examine changes in capillary compliance. Henceforth, the various salient features of this model are presented.

### The “capillary-tissue” computational system

**Fig 1:**
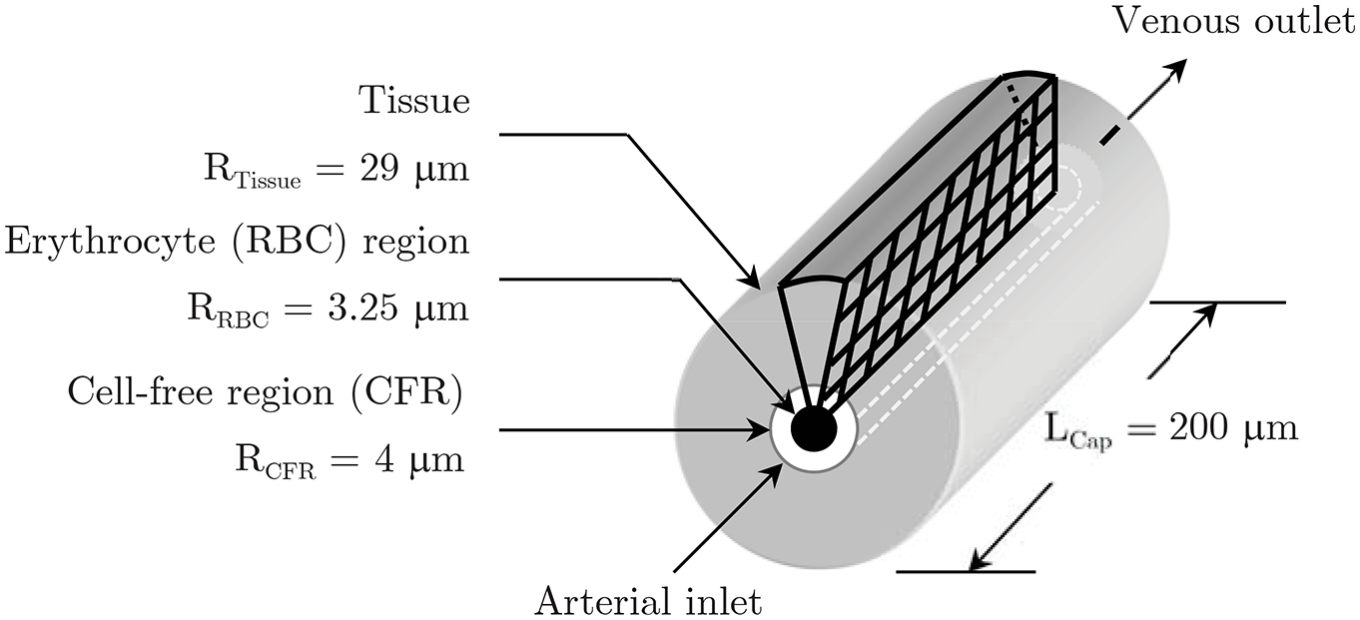
The capillary-tissue computational system and the axisymmetric computational grid plane. The capillary, subdivided into an erythrocyte and a cell-free plasma zone, feeds a uniform thickness tissue supply zone. Fig 2 shows part of the computational grid in more detail.

**Fig 2:**
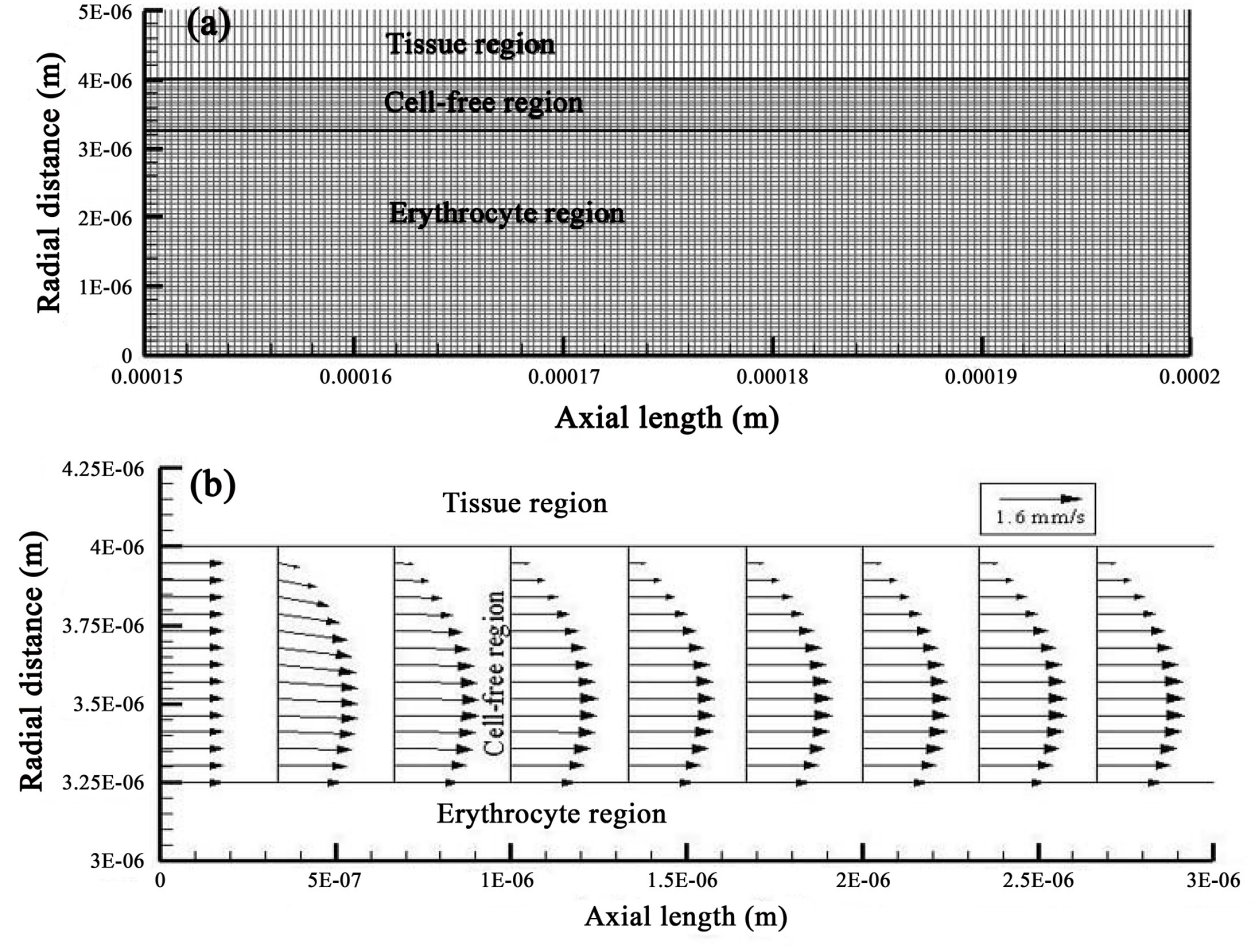
Computational implementation and spatially resolved velocity results for the capillary-tissue system. Upper figure (a) shows a detailed view of the computational mesh of the capillary-tissue model (depicted in Fig 1) for a capillary length of 150 to 200 *μ*m and radius of 5 *μ*m. The mesh consists of 34,038 cells. Lower figure (b) displays the developed velocity field, for a CMR_O_2__ of 250 *μ*mol/100g/min and a CBv of 1.2 mm/s (case H), at the arterial inlet of the plasma region.

**Fig 3:**
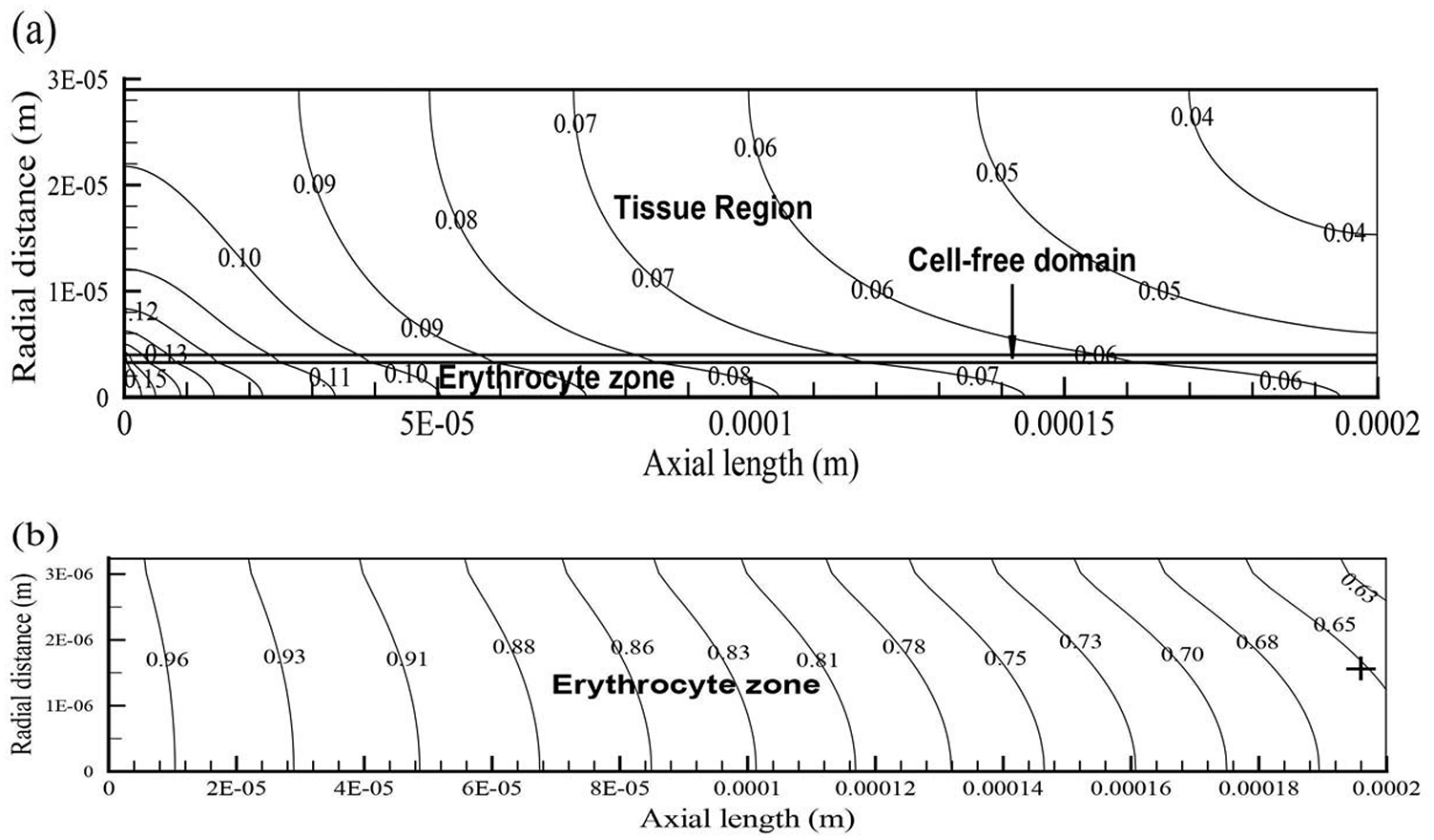
Oxygen levels and hemoglobin saturation contours of the capillary-tissue system. Figure (a) displays the oxygen isoconcentration contours in the capillary and in the cortical tissue for CMR_O_2__ = 250 *μ*mol/100g/min and CBv = 0.9 mm/s 3 s after the increase in CBv. Figure (b) shows the distribution of hemoglobin saturation in the RBC region for the abovementioned CMR_O_2__ and CBv. Hemoglobin saturation (sO_2_) values, referred to in the manuscript, were probed at the rightmost side of the RBC domain marked by the cross.

The computational model (Fig 1) consists of a straight capillary, 8 *μ*m in diameter by 200 *μ*m in length, subdivided into an inner erythrocyte and an annular cell-free region (CFR) measuring 3.25 *μ*m in radius and 0.75 *μ*m thick, respectively. Consisting of a single cell wall, the capillary endothelium was taken to possess the same metabolic characteristics as those of the tissue. The cerebral capillary perfuses a uniform thickness tissue region extending to 29 *μ*m [22]. Intercapillary spacing derives from the observation that functional cells reside no more than 30 *μ*m away from a capillary [39], hence the tissue supply region was set to 25 *μ*m. Fig 2(a) and 2(b) show, in detail, part of the computational model presented in its entirety in Fig 3. A finer mesh in the erythrocyte (7,686 cells) and the cell-free (2,196 cells) domains, relative to the tissue zone (24,156 cells), ensures the accuracy of results is not compromised. Unlike the large distribution, in the literature, of erythrocyte velocity values (Table 1), region-specific oxygen diffusion coefficient (D_O_2__) values exhibited small variability. To minimize the former variability we have examined a broad range of basal and activated blood velocities and metabolic rates, and assessed their interaction and influence against physiological hemoglobin saturation values.

**Table 1:** Tissue-capillary system particulars.

Parameter	Definition	Source
L_RBC_ = 200 *μ*m	RBC region length	[20]
L_CFR_ = 200 *μ*m	Cell-free region length	[20]
L_Tissue_ = 200 *μ*m	Cerebral tissue length	[20]
R_RBC_ = 3.25 *μ*m	RBC (erythrocyte) region radius	[21]
t_CFR_ = 0.75 *μ*m	Cell-free region (CFR) thickness	[21]
t_Tissue_ = 25 *μ*m	Brain tissue thickness	[22]
*ρ*_RBC_ = 1,160 kg/m^3^	RBC mass-density	[23]
*ρ*_CFR_ = 1,025 kg/m^3^	Cell-free region (plasma) mass-density	[24]
*ρ*_Tissue_ = 1,040 kg/m^3^	Brain tissue mass-density	[25]
*μ*_CFR_ = 1.2 × 10^−3^ kg/m-s	Cell-free region dynamic viscosity	[23]
D_O_2_RBC___ = 8.8 × 10^−6^ cm^2^/s	RBC O_2_ diffusivity	[26, 27]
D_O_2_CFR___ = 2.29 × 10^−5^ cm^2^/s	Cell-free region (plasma) O_2_ diffusivity	[13, 28]
D_O_2_Tissue___ = 1.6 × 10^−5^ cm^2^/s	Brain tissue O_2_ diffusivity	[27]
D_Hb_ = 1.42 × 10^−7^ cm^2^/s	RBC hemoglobin diffusivity	[13, 29]
CBv = 0.3–1.2 mm/s	Capillary blood velocity	[30–32]
CMR_O_2__ = 200–466^i^	Metabolic rate of O_2_ utilization	[27, 33, 34]
*c*_H_ = 9.1 mol/m^3^	Oxygen carrying capacity of Hb	[32]
*k*_H_ = 40 s^−1^	Reaction rate of HbO_2_ dissociation	[35]
$H_{{\text{O}}_{2}}=769.2\text{L-atm/mol}$	Henry’s law (oxygen) constant	[36]
$n=\frac{\Delta \text{CBF}/\text{CBF}}{\Delta {\text{CMR}}_{{\text{O}}_{2}}/{\text{CMR}}_{{\text{O}}_{2}}}$	Normalized ΔCBF to ΔCMR_O_2__	–
$m=\frac{\Delta \text{CBv}/\text{CBv}}{\Delta {\text{CMR}}_{{\text{O}}_{2}}/{\text{CMR}}_{{\text{O}}_{2}}}$	Normalized ΔCBv to ΔCMR_O_2__	This study
H_T_ = 0.31	Tube hematocrit	[37]

^i^Units: *μ*mol/100g/min.

While some authors neglect axial diffusion [32], others quantitatively justify its importance [40]. In this study both convection and diffusion are considered in the axial and radial directions of the capillary-tissue system. The computational model presented in this work was developed using the multi-physics CFD-ACE+ platform (ESI, Paris, France) [41].

#### Erythrocyte (RBC) Region

Experimental measurements indicate that the migration of hematocytes towards the center of small diameter blood vessels (e.g., capillaries) results in a cell-depleted zone, adjacent to the endothelial wall, with a thickness on the order of a few microns [42]. Extrapolating from these findings the RBC region was approximated to occupy a radius of 3.25 *μ*m. This innermost RBC region was assumed to undergo a solid-type translational motion exhibiting a uniform velocity, accounting for the nature of RBC transport. The rationale of adopting a continuous RBC region stems from observations in comparable diameter glass tube experiments where erythrocytes often aggregate to form continuous packets [42]. Readers interested in the mechanics of erythrocyte transit through microvessels, which lies beyond the scope of this investigation, may consult relevant sources [21, 43].

For all computations, a tube hematocrit of 0.31 was maintained [37] (a range of values was tested; results not reported in here). Tube hematocrit (H_T_) refers to the volume fraction of suspended red blood cells in a microvessel (tube). For an erythrocyte mean cell volume (MCV) of 90 fL (10^−15^L) and an H_T_ level of 0.31, the capillary’s erythrocyte cell count amounts to about 23 corpuscles. Oxygen release and the concomitant drop in hemoglobin saturation were governed by the non-linear sigmoidal shape Oxygen-Hemoglobin Dissociation Curve (OHDC) [44]. Hemoglobin flux through the RBC inlet, at a pO_2_ of 93.4 mmHg, was 97% saturated. Oxygen and hemoglobin convection and diffusion were accounted for, by solving the general transport equation for oxygen partial pressure, hemoglobin saturation and their interaction.

#### Cell-Free Region (CFR)

In this region, the equations of fluid flow (Eqs (1), (2a) and (2b)) were solved, together with the general transport Eq (3) which collectively defined oxygen convection and diffusion. The fully developed flow assumed a parabolic velocity profile, appropriately adjusted to satisfy the imposed boundary conditions. Oxygen flux, from the 0.75 *μ*m thick RBC-free region, into the tissue domain, was influenced by the combined effects of convection and diffusion.

#### Tissue Region

During rest and activation, cortical tissue, nourished by the capillary, was assumed to consume oxygen at a uniformly distributed rate throughout the cortical tissue. Oxygen transport in the tissue was governed by the general transport Eq (3), with only diffusive transport being active. Extracellular effects of neuroglobin-facilitated diffusion [14] were accounted for by the tissue oxygen diffusion coefficient. For the sake of simplicity, the single cell-thick capillary endothelial wall was taken to possess the same metabolic characteristics as those of the tissue. Oxygen metabolism, which is a function of molar tissue oxygenation, was implemented as a sink term (−*S*_*ϕ*_*i*__) in Eq (3).

### Governing equations

The Navier-Stokes and mass conservation equations were solved to model the behavior of the plasma in the CFR while the inner RBC region was prescribed a uniform velocity (supplying thus no-slip, moving layer boundary conditions for the plasma). In cylindrical polar coordinates (*r*, *θ*, *z*), where *r* is the radial distance, *θ* the (azimuth) angle, and *z* the distance from the chosen plane to the point of interest, in the absence of a circumferential velocity component (*u*_*θ*_ = 0), the continuity equation reduces to:

$$\begin{array}{c} \frac{1}{r}\frac{\partial (ru_{r})}{\partial r}\;+\;\frac{\partial u_{z}}{\partial z}\;=\;0\;. \end{array}$$ (1)

Under axisymmetric conditions (*u*_*θ*_ = 0) the Navier-Stokes equations become:

$$\begin{array}{c} \rho \left(\frac{\partial u_{r}}{\partial t}+u_{r}\frac{\partial u_{r}}{\partial r}+u_{z}\frac{\partial u_{r}}{\partial z}\right)\;=\;-\frac{\partial p}{\partial r}+\mu \left(\frac{1}{r}\frac{\partial }{\partial r}\left(r\frac{\partial u_{r}}{\partial r}\right)+\frac{\partial^{2}u_{r}}{\partial z^{2}}-\frac{u_{r}}{r^{2}}\right) \end{array}$$ (2a)

$$\begin{array}{c} \rho \left(\frac{\partial u_{z}}{\partial t}\;+\;u_{r}\;\frac{\partial u_{z}}{\partial r}\;+\;u_{z}\;\frac{\partial u_{z}}{\partial z}\right)\;=\;-\frac{\partial p}{\partial z}\;+\;\mu \;\left(\frac{1}{r}\;\frac{\partial }{\partial r}\;\left(r\frac{\partial u_{z}}{\partial r}\right)\;+\;\frac{\partial^{2}u_{z}}{\partial z^{2}}\right)\;. \end{array}$$ (2b)

where *p* is pressure and *μ* the fluid viscosity. Although it is expected that the scale of the geometric configuration explored would make the inertial terms in the Navier-Stokes equations small in magnitude, we chose to include them (at some additional computational cost) in casting of the governing equations. The flow field is solved using the Semi-Implicit Method for Pressure-Linked Equations Consistent (SIMPLEC) algorithm [45]. Hemoglobin transport, within the RBC region, and oxygen efflux from the same domain into the CFR and eventually into tissue were governed by appropriate mass transport equations for each species. The general transport equation in cylindrical polar coordinates, for an axisymmetric configuration, is:

$$\begin{array}{c} \frac{\partial \phi_{i}}{\partial t}+\frac{u_{r}}{r}\frac{\partial }{\partial r}\left(r\;\phi_{i}\right)+u_{r}\frac{\partial \phi_{i}}{\partial z}=\Gamma_{ij}\left(\frac{1}{r}\frac{\partial }{\partial r}\left(r\frac{\partial \phi_{i}}{\partial r}\right)+\frac{\partial^{2}\phi }{\partial z^{2}}\right)+S_{\phi_{i}}\;. \end{array}$$ (3)

where *u*_*r*_ is the flow velocity in the *r* direction, Γ_*ij*_ is the diffusivity coefficient of species *i* in spacial domain *j*. Scalars *ϕ*_*i*_ denote the dependent variables of hemoglobin and oxygen. The derivation of the OHDC reflects the complex chemical reaction kinetics of the combination and dissociation between oxygen and hemoglobin. The significant biological importance of the OHDC derives from the remarkable ability of the mammalian oxygen bound hemoglobin not to directly contribute to blood pO_2_ thus enhancing the blood’s oxygen carrying capacity by 30 to 50 fold, in relation to the freely dissolved plasma oxygen [5]. Oxygen release and oxyhemoglobin dissociation in the erythrocyte region appear as source/sink terms (*S*_*ϕ*_*i*__), which derive from the hemoglobin reaction rate [29, 40]:

$$\begin{array}{c} {\text{R}}_{\text{Hb}}=k_{\text{H}}\cdot c_{\text{H}}\cdot \left(\frac{{\text{sO}}_{2\;\text{Num}}-{\text{sO}}_{2\;\text{OHDC}}}{1-{\text{sO}}_{2\;\text{OHDC}}}\right)\;. \end{array}$$ (4)

where *k*_H_ is the reaction rate of oxyhemoglobin desaturation and *c*_H_ is the oxygen carrying capacity of hemoglobin (see Table 1). Fractional oxyhemoglobin saturation sO_2Num_ and sO_2OHDC_ values were determined quasi-numerically, using the OHDC, and numerically, respectively. For an implementation of the OHDC, as part of the detailed procedure, not presented herein one may consult reference [19]. Naturally, the tissue cannot consume more oxygen than it is physically and spatially present at any instant in time.

### Oxygen metabolic and blood velocity transitions

To determine the existence of possible non-linearities in the BOLD effect, we assessed the impact that neuronal activation, in the context of CMR_O_2__ and CBv transitions, exerts on the fractional hemoglobin saturation at the venous outlet of the capillary. Using mass conservation, the deoxygenation proportion of hemoglobin can be deduced from the degree of blood oxygenation. A positive BOLD signal is obtained when the level of oxyhemoglobin increases such that it displaces deoxyhemoglobin which has been suppressing the MR signal. Our analysis is based on the hypothesis that increases in blood flow reflect cerebral metabolic transitions. Coupling of blood flow and CMR_O_2__ is maintained through blood velocity changes into the perfused capillary.

Nearly all studies [2, 6, 8, 11, 46, 47] which involve the use or study of blood flow and brain oxygen metabolism (CMR_O_2__) utilize cerebral blood flow (CBF). Normalized changes in blood flow (in units of mL/100g/min) and oxygen metabolism (in units of *μ*mol/100g/min) during activation are expressed by the simple ratio of (ΔCBF/CBF)/(ΔCMR_O_2__/CMR_O_2__), often indicated by *n*. However, for microscopic capillary modeling a more appropriate metric to use is the fractional change in blood flow velocity (mm/s) to oxygen metabolism: (ΔCBv/CBv)/(ΔCMR_O_2__/CMR_O_2__), herein defined as *m*. Distinctly from early PET investigations by Fox and Raichle [15] and Fox *et al*. [16], several authors [47–50] report changes in *n* ranging from 2 to 5. CBv values, retrieved from various sources, which ranged from 0.3 mm/s to 1.2 mm/s, exhibited considerable variability in relation to CMR_O_2__ values, for grey matter, which varied by 133% (Table 1).

**Fig 4:**
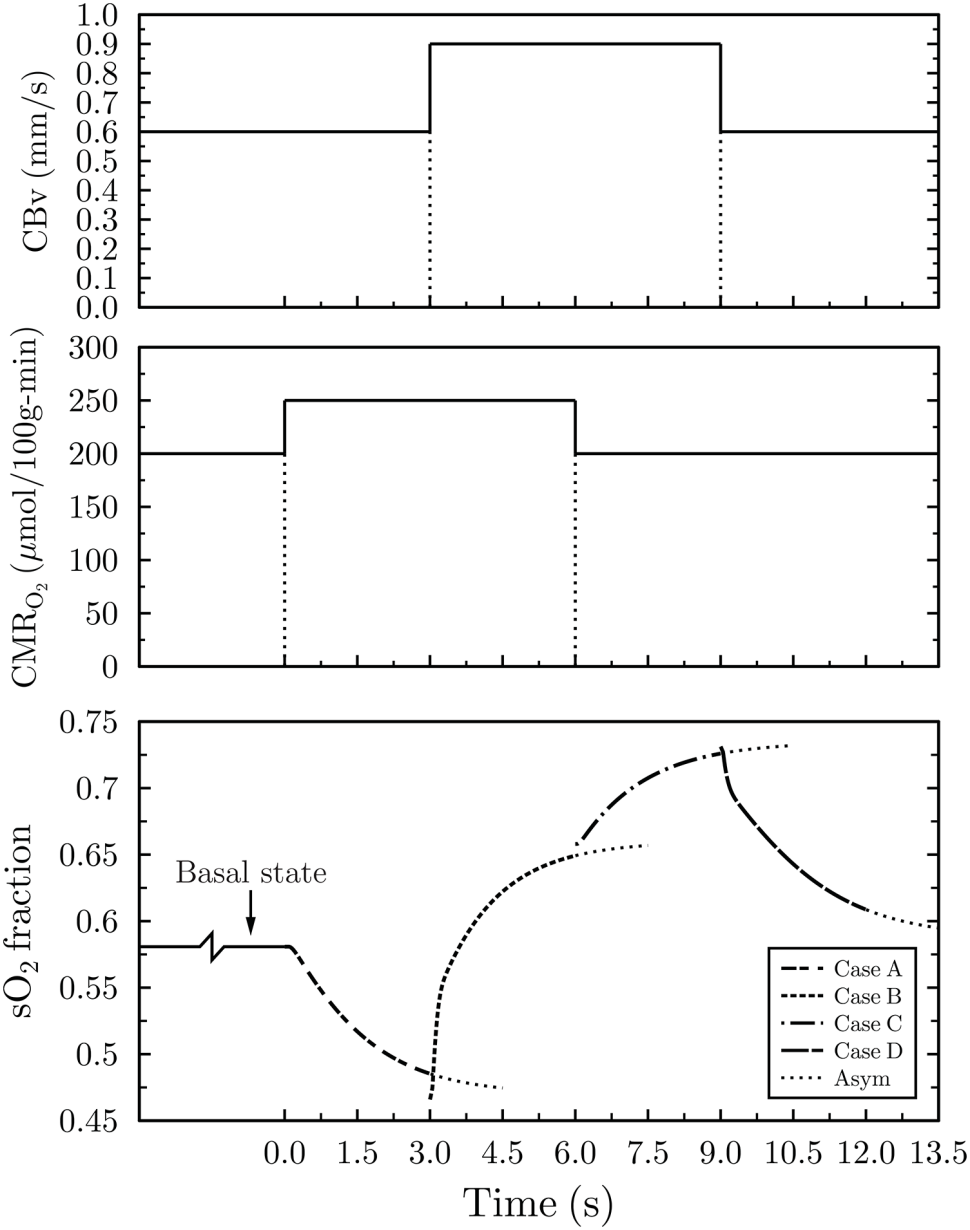
Hemoglobin saturation response at the capillary outlet for *m* = (ΔCBv/CBv)/(ΔCMR_O_2__/CMR_O_2__) = 2. The lower plot depicts hemoglobin saturation following neural activation, mimicked by the four discrete physiological cases, namely, A to D. Once stimulation occurs, CMR_O_2__ increases, from its resting (basal) state, from 200 to 250 *μ*mol/100g/min (case A) causing a drop in sO_2_. Subsequently, CBv increases from 0.6 to 0.9 mm/s resulting in a steep increase in saturation (case B). Next, CMR_O_2__ reverts back to its basal level of 200 *μ*mol/100g/min leading to a further increase in sO_2_ (case C). Finally, CBv transitions to its resting state (case D) with the concomitant asymptotic drop in sO_2_ to its basal value. The top and middle plots illustrate the duration of the activated and basal oxygen metabolic and blood velocity transitions, respectively. “Asym” stands for asymptotic values.

Experimental evidence indicates that changes in CMR_O_2__ are much smaller than variations in blood flow [15]. To verify the latter observation as well as to determine the influence other factors, such as neurovascular coupling, under steady state and transient conditions, have on the BOLD effect (through changes in the venous hemoglobin saturation) we have formulated a number of physiological scenarios outlined in the last part of this section and summarized in Table 2. A basal CMR_O_2__ of 200 *μ*mol/100g/min, typical value for the human cortex [33] and a CBv of 0.6 mm/s were used, for *m* = 2, 3 & 4. Tracing neuronal activity, CMR_O_2__ was assumed to undergo a 25% increase to 250 *μ*mol/100g/min— in line with values reported by Marrett and Gjedde [47]— followed by an amplification in basal CBv from 0.6 mm/s, to 1.05 mm/s, to 1.2 mm/s for scenarios I, II, and III, as shown in Table 2. Detailed features of the regional in vivo oxygen utilization characteristics, like oxygen metabolic rates in humans, during activation, at the capillary scale were difficult to identify in the literature. Therefore, CMR_O_2__ and CBv transitions, at *t* = 0 s, were considered as step changes, as seen in Fig 4, from the previous’ case asymptotic results.

**Table 2:** Time constants (*τ*) for scenarios I, II and III comprising cases A to J, for *m* = 2, 3 & 4. Each scenario is based either on an oxidative metabolic change or a blood velocity transition in each case spanning over 3 s plus 1.5 s of asymptotic behavior.

$m(\frac{\Delta \text{CBv}/\text{CBv}}{\Delta {\text{CMR}}_{{\text{O}}_{2}}/{\text{CMR}}_{{\text{O}}_{2}}})$	Scenario	Case	CMR_O_2__ (*μ*mol/100g/min)	CBv (mm/s)	Time constant^i^ *τ* (s)
2, 3, 4	I, II, III	A	200 to 250	0.6	1.395
2	I	B	250	0.6 to 0.9	0.623
		C	250 to 200	0.9	1.206
		D	200	0.9 to 0.6	1.187
3	II	E	250	0.6 to 1.05	0.171
		F	250 to 200	1.05	0.391
		G	200	1.05 to 0.6	0.421
4	III	H	250	0.6 to 1.2	0.147
		I	250 to 200	1.2	0.381
		J	200	1.2 to 0.6	0.449

^i^The time constant *τ* is the time duration for sO_2_ to reach 1 − *e*^−1^ of its final value for the time course of CBv or CMR_O_2__ transitions.

Due to its significance to the BOLD contrast, temporal hemoglobin saturation was probed at the capillary outlet, for each of the four physiological states described below. For each consecutive case only one physiological parameter, either CMR_O_2__ or CBv was varied at a time (except for neurovascular coupling), while each case used the asymptotic findings of the previous case as its starting point. In quantifying the sensitivity of the system (model) to physiological changes a broad range of flow velocities and oxidative metabolic rates, constituting *m* = 2, 3 & 4, were considered on purpose. Furthermore, the temporal and physiological characteristics of neurovascular coupling effects were examined. Subsequent sections outline the cases for scenarios I, II, and III, whose characteristics are listed in Table 2.

#### Activated CMR_O_2__, resting CBv

Triggered by neural activity, the basal CMR_O_2__ of 200 *μ*mol/100g/min was increased, by 25%, to 250 *μ*mol/100g/min, while CBv remained unaltered to 0.6 mm/s.

#### Activated CMR_O_2__, activated CBv

The influence of cerebral activation on CBv was modeled as velocity transitions from 0.6 mm/s to 0.9 mm/s, an increase of 50%, to 1.05 mm/s, a surge of 75%, and 1.2 mm/s (an augmentation of 100%) for consecutive *m* changes of 2, 3, and 4, respectively.

#### Resting CMR_O_2__, activated CBv

Assuming a shorter stimulus duration, relative to the time scale of vascular adaptations, CMR_O_2__ can revert back to its basal state while CBv remains elevated. Hence, CMR_O_2__ will drop to 200 *μ*mol/100g/min and blood velocity will continue to perfuse the capillary at the increased value of 0.9 mm/s, or 1.05 mm/s, or 1.2 mm/s for each distinct case.

#### Resting CMR_O_2__, resting CBv

Finally, once the stimulus has been removed and blood velocity has subsided to its basal value, the system resorts to its resting (equilibrium) state CBv of 0.6 mm/s (and CMR_O_2__ of 200 *μ*mol/100g/min).

## Results

All computational results obtained from the capillary-tissue system (Fig 2) are second order accurate in space and time. As illustrated by the velocity vector field of case H (Fig 2), with the highest velocity of 1.2 mm/s, plasma entering the capillary assumes its fully developed form shortly after its entry in the cell-free region (1 *μ*m past the entrance). Influenced by the upper (tissue boundary) and lower (erythrocyte boundary) no-slip conditions of 0 mm/s and 1.2 mm/s, respectively, the steady inlet velocity of 1.2 mm/s develops into a parabolic-like profile for plasma. Thereafter, CBv retains its skewed parabolic pattern throughout the entire capillary length. Predominantly influenced by CBv and oxygen consumption in the tissue, oxygen in the capillary domain traverses the capillary at a faster rate than its pace of penetration into the tissue (Fig 3). Hemoglobin saturation (sO_2_), probed at the capillary outlet (cross sign), exhibits a similar pattern also observed throughout the erythrocyte region (Fig 3).

### Hemoglobin response profile for *m* = 2

Temporal mapping of the hemoglobin saturation fraction, at the capillary outlet, for *m* = 2, produced a hemoglobin profile within physiological limits, and in excellent agreement with other research reports [2, 6, 51]. It is important to emphasize that these theoretical results, based on the fundamental physical and physiological mechanisms believed to govern the BOLD phenomenon, were obtained from first principles with the least number of assumptions possible.

As depicted in Fig 4, for *m* = 2, the basal CBv of 0.6 mm/s and CMR_O_2__ of 200 *μ*mol/100g/min resulted in a terminal fractional hemoglobin saturation of 0.58 (Fig 4, Basal state). Terminal and peak Oxygen Extraction Fraction (OEF) or sO_2_ refer to the final OEF (or sO_2_) for the 3 s duration of each case and not of their asymptotic values. Utilizing the arterial (sO_2,*a*_) and venous (sO_2,*ν*_) hemoglobin saturation fractions, derived from the model, the OEF can be determined from:

$$\begin{array}{c} \text{OEF}=\frac{{\text{sO}}_{2,\;a}-{\text{sO}}_{2,\;\nu }}{{\text{sO}}_{2,\;a}} \end{array}$$ (5)

Thus the basal state sO_2_ of 0.58 yields an OEF of 0.4 which turns out to be exactly the same with the baseline OEF found in other reports [2, 6, 7, 51]. On the other extreme, for the case of activated velocity, that is, CBv of 0.9 mm/s and CMR_O_2__ of 200 *μ*mol/100g/min, sO_2_ reached 0.73 (Fig 4, Case C) which translates into an OEF of 0.25.

For case C (resting oxygen utilization and activated velocity) and *m* = 2, results lie within physiological limits (Fig 4). Marchal *et al*. [51] report human resting oxygen extraction fractions ranging between 35–55% while Buxton *et al*. [7] cite an activated OEF of 0.3. Although case C (Fig 4) with a peak OEF of 0.25 appears to slightly underestimate the OEF value quoted in Buxton *et al*. [7], the relative timing of physiological changes can fundamentally alter the magnitude of the OEF. Likewise, the drop in hemoglobin saturation for case A is intrinsically tied to the temporal characteristics of the neurogenic control which brings about the increase in CBv. The shorter the elapsed time between the increase in oxidative metabolism and the arrival of increased velocity blood, the smaller the drop in hemoglobin saturation. A time lag between the two physiological processes supports the occurrence of the initial dip, which will be elaborated upon in subsequent sections.

### Hemoglobin response profile for *m* = 3 & *m* = 4

**Fig 5:**
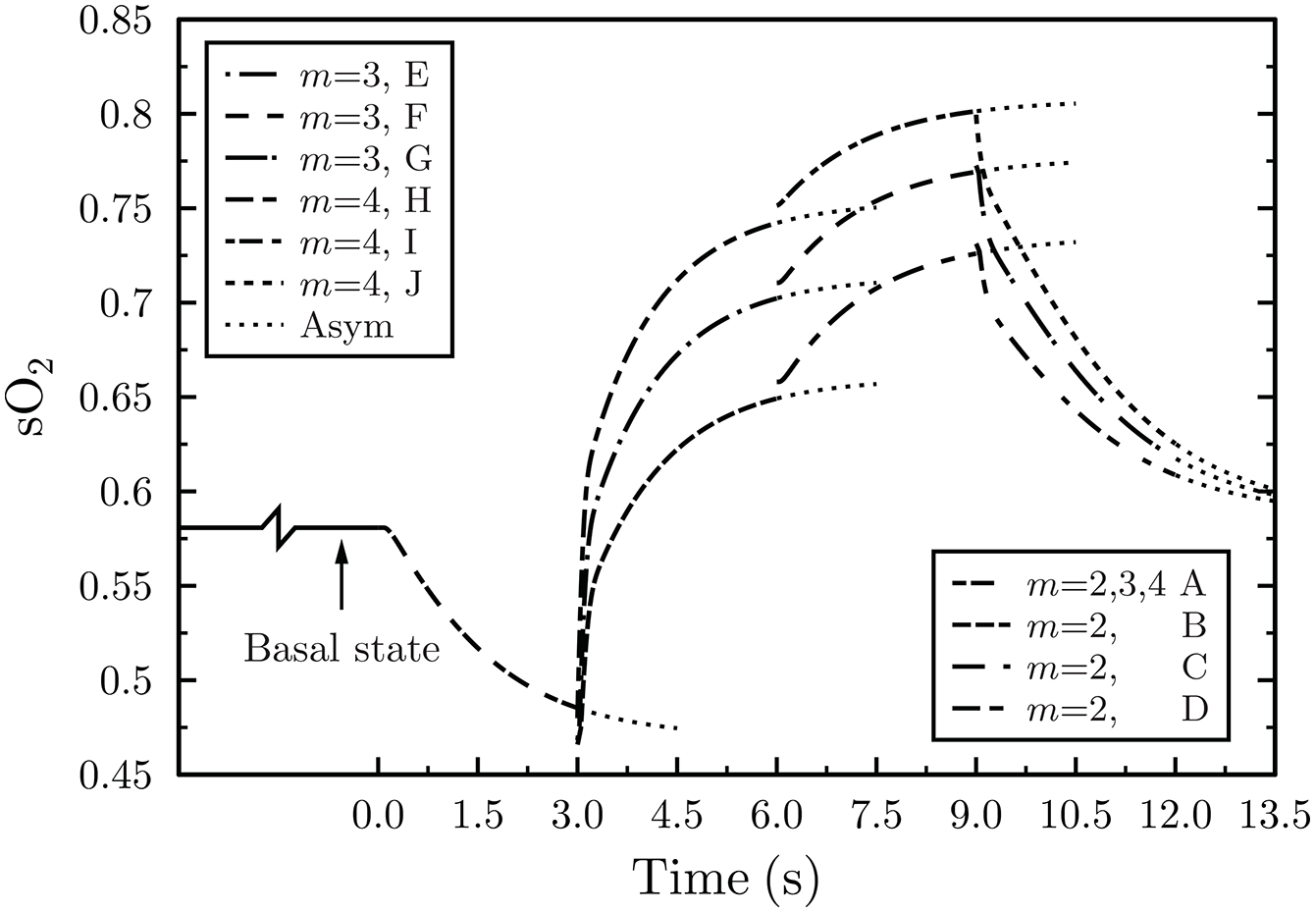
Hemoglobin saturation response, at the capillary outlet, for *m* = 2, 3 and 4. Cases A, B, C, and D are the same as those depicted in Fig 4. “Asym” denotes asymptotic values.

To test the range of *m* values, for grey matter, which yield results within physiological limits and to assess the sensitivity of the system to physiological changes, in addition to *m* = 2, *m* = 3 and *m* = 4 were considered, as illustrated in Fig 5. Increasing the *m* value from 2 to 3 and finally to 4 raises the sO_2_ profile. Case E, predicated on a boost in CBv from 0.6 mm/s to 1.2 mm/s, results in a dramatic increase in sO_2_. Upon deactivation, a drop in CMR_O_2__ from 250 to 200 *μ*mol/100g/min (case F) elevates end sO_2_ above physiological boundaries. Finally, when CBv drops from 1.05 mm/s to its basal value of 0.6 mm/s, hemoglobin saturation returns to its resting level (case G). A similar trend is observed for cases H, I, and J. Case H, with the largest increase in CBv, from 0.6 mm/s to 1.2 mm/s, produces the swiftest rise in sO_2_. This response reflects the sensitivity of the system to blood velocity changes, rather than changes in CMR_O_2__, and demonstrates the influence of convective transport on hemoglobin saturation.

For the activated CBv = 1.05 mm/s and basal CMR_O_2__ of 200 *μ*mol/100g/min for case F, scenario II, as depicted in Fig 5, terminal hemoglobin saturation climbs to 0.77 and OEF drops to 0.21; a value commensurate to the OEF of 0.3 used in Buxton *et al*. [7]. Correlating the basal and increased velocity cases of 250 *μ*mol/100g/min and CBv of 0.9 mm/s (case B, Fig 5) and 1.2 mm/s (case H, Fig 5) it becomes evident that sO_2_ and OEF are more responsive to changes in blood velocity than oxygen consumption. Coherently, changes in blood velocity have a greater effect on the hemoglobin oxygenation gradient across the capillary than variations in the magnitude of tissue oxygen consumption. This is partly attributed to the aptitude of convection for long distance transport and the slower diffusive tissue time scales arising from diffusion’s deficiency associated with large distance oxygen transfer. Depending on the saturation level of hemoglobin, driven by the pO_2_, the ease (or difficulty) of oxygen release, governed by the OHDC, is anticipated to influence the capillary outlet sO_2_. Faster oxygen release is attained when the OHDC rate of change is very steep. Hemoglobin saturation experiences its greater drop in the 10 to 60 mmHg (pO_2_) range where oxygen dissociation is pronounced.

The system’s responsiveness to physiological transitions is quantitatively captured by the time constant (*τ*), herein defined to be the time it takes for hemoglobin saturation to reach 63.2% of its final value. In other words, *τ* reflects the responsiveness of sO_2_ (and OEF) to perturbations in velocity or oxygen metabolism. For *m* = 2, 3 & 4, it was found that sO_2_ responded faster to changes in CBv than CMR_O_2__ transitions. Indeed, the most dramatic response is observed when CBv is increased from its resting to its activated level. Transitions in CBv from 0.6 mm/s to 0.9 mm/s (case B) to 1.05 mm/s (case E) and 1.2 mm/s (case H) yield the shortest time constants (*τ*) within a particular *m* value for each scenario, respectively. As shown in Table 2, scenario I, case B, produces a *τ*_*m* = 2_ of 0.623 s, case E, scenario II, yields a *τ*_*m* = 3_ value of 0.171 s, and scenario III, case H, generates a *τ*_*m* = 4_ of 0.147 s. In turn, the drastic decreasing trend in *τ*, across *m* values, for cases B, E, and H, emanates from the precipitous rate of change in sO_2_.

In contrast, the metabolic activation shift from 200 to 250 *μ*mol/100g/min (case A), as indicated by the highest *τ* value of 1.395 s (Table 2), produces the slowest sO_2_ change. Notably, cases C, F, and I, which undergo the same drop in CMR_O_2__ from 250 to 200 *μ*mol/100g/min, despite their higher CBv of 0.9 mm/s (case C), 1.05 mm/s (case F), and 1.2 mm/s (case I) the shape of their sO_2_ curves (depicted in Fig 5) remained fundamentally unaltered. Similarly, cases D, G, J which share the same CMR_O_2__ of 200 *μ*mol/100g/min even though they possess different initial CBv, that is, 0.9 mm/s (case D), 1.05 mm/s (case G), and 1.2 mm/s (case J) when they resort to their basal CBv value of 0.6 mm/s they yield curves of largely comparable shape.

### Reproducing the sO_2_ profile— A marker for the initial dip

Considering the complex interplay between the physical and physiological mechanisms thought to be responsible for the BOLD response, it is improbable that physiological changes in CMR_O_2__ and CBv occur as discrete events. For this investigation, scenario I (comprising cases A to D) was selected because it generates terminal sO_2_ values within physiological limits. There is accumulating evidence [52–54] to suggest that the hemodynamic response commences ≈2 s after stimulus onset, with a noticeable peak at about 5–6 s, while the entire response lasts between 8–10 s. The temporal dynamics of changes in CBv and CMR_O_2__ modeled here can potentially alter both the magnitude and duration of the hemoglobin saturation response from which the BOLD effect derives.

**Fig 6:**
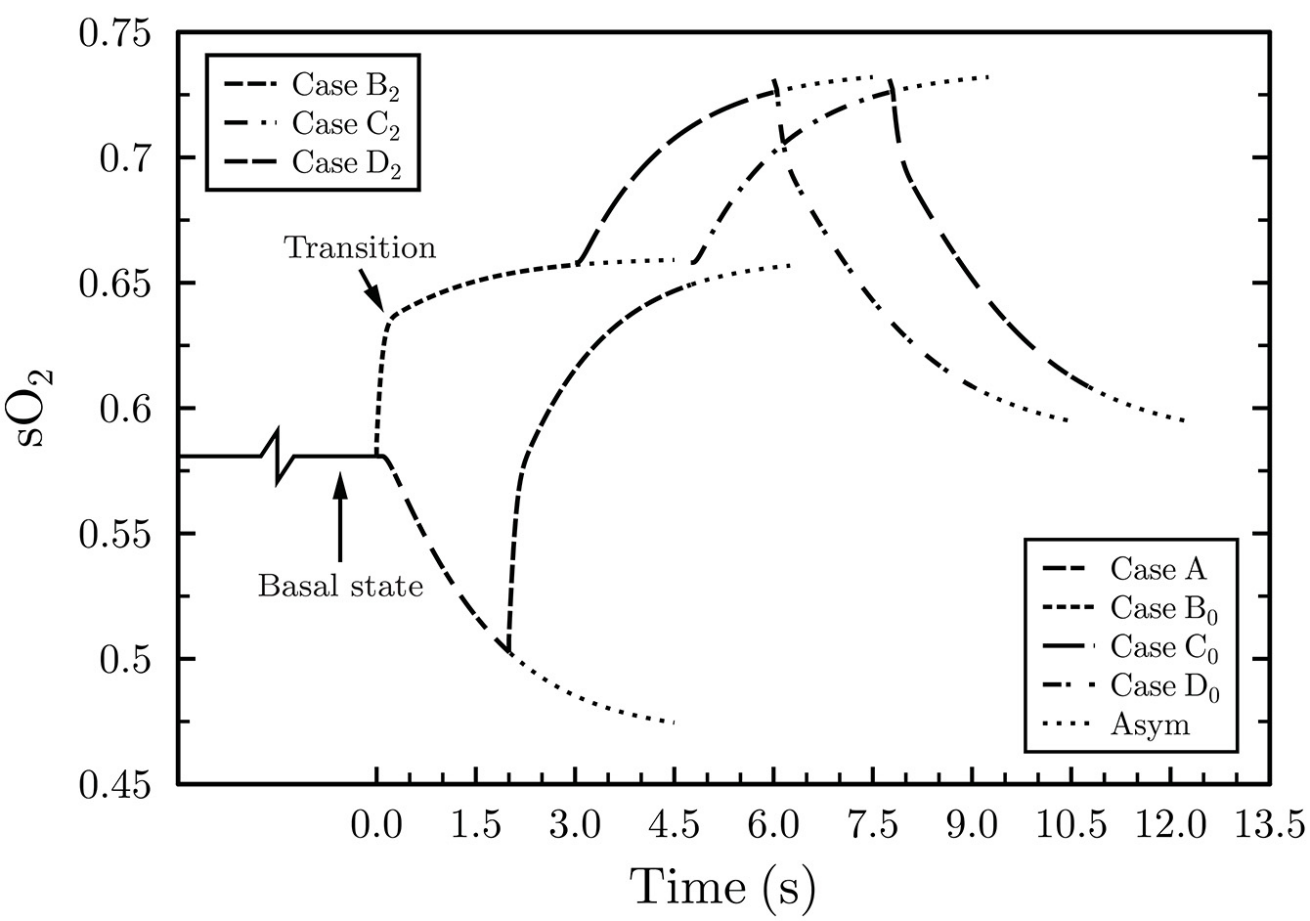
Capillary outlet hemoglobin saturation response, for *m* = (ΔCBv/CBv)/(ΔCMR_O_2__/CMR_O_2__) = 2, considering neurovascular effects. Case B_0_ assumes no time lag between the latter physiological mechanisms but case B_2_ is predicated on a 2 s delay after the onset of metabolism.

Accordingly, the shape of the sO_2_ profiles (Fig 6) and the upper and lower sO_2_ levels are governed by the time coherence and duration of transitions in CMR_O_2__ and CBv. These same changes can be held accountable for the manifestation (or hindrance) of the initial dip. However, the neurovascular mechanism and the time lag between neuronal activation and increase in CBv, believed to the linked to the initial dip, remain obscure. In this report we shed light on a specific aspect of the BOLD initial dip and, in particular, the temporal dynamics of cerebral blood flow. More details about the initial dip are included in the Discussion section. It is impossible for blood reaching an activated brain region to arrive from too distal a location to the epicenter of activation within a very short period of time. The major difference between the sO_2_ curves in Figs 4 and 6 (bottom plot) lies in the time lag between the increase in CBv in relation to the activated CMR_O_2__.

Considering the delay in the arrival of increased CBF, following neuronal activation, which ranges from 0–2 s (Buxton *et al*. [7]) and the physiological limits of hemoglobin saturation during rest and activation, the timing of physiological changes for *m* = 2 was adjusted accordingly. Because it can fundamentally alter the extent and shape of the sO_2_ profile, the time lag of the increased velocity blood, that accompanies activation, is critically important. Earlier research [3, 55], which concentrated on the cortical tissue of animal models, dealt with the physiological changes and time scales of cerebral metabolic responses. Yet the temporal specificity of the arrival of increased velocity blood following neuronal activation remains the subject of further research. Therefore, for *m* = 2, we have investigated the two extreme scenarios for increased CBv reaching the capillary inlet.

For the first one, the increase in blood velocity was taken to coincide with the increase in CMR_O_2__, viz, at *t* = 0 s (case B_0_, Fig 6), signifying no time delay. Second, the elevated CBv was assumed to lag 2 s behind metabolic activation (case B_2_). Although cases B_0_ and B_2_ produce the same end sO_2_ values, the different CBv onset times mean that the increased CBv encounters different initial conditions. When the timing of the elevated CMR_O_2__ and CBv overlap, as indicated by the initial capillary sO_2_ of 0.58, the tissue is already replete with oxygen. However, the 2 s mismatch, during case B_2_, causes sO_2_ to drop to 0.5 reflecting a much lower tissue oxygen concentration. Hence, the difference in tissue oxygen content is, predominantly, held responsible for the dissimilar rates of change of the sO_2_ of curves B_0_ and B_2_ (Fig 6).

At the beginning, the two sO_2_ curves display virtually identical dramatic saturation changes, however, soon an instant (in time) is reached beyond which the rate of increase of sO_2_ flattens. For B_0_ the latter transition occurs 0.022 s after CBv increases (labeled “Transition” on Fig 6), which is the time it takes for saturated blood, entering the capillary at *t* = 0 s, to traverse the entire capillary. Due to the lower initial tissue oxygen content, during case B_2_ (Fig 6), the sO_2_ curve remains predominantly steep for the entire duration of the physiological change. In terms of responsiveness, B_0_ yields a time constant *τ*_0_ = 0.051 s while B_2_ generates a *τ*_2_ = 0.198 s, hence B_0_ reaches 63.2% of its final sO_2_ level almost four times faster than B_2_.

Another important consequence of neurovascular coupling is its impact on the overall duration of the hemoglobin saturation profile (Fig 6). If the hemodynamic signal were to peak 5–6 s after the onset of activation, then this permits a certain degree of temporal overlap between cases A, B_2_, and C_2_. Possible non-linearities in metabolism and blood velocity could also alter the temporal characteristics of the sO_2_ profile and the manifestation of the initial dip. Although when originally observed the negative dip appeared particularly promising in terms of localizing neuronal activity [3, 56] yet its inconsistency across subjects remains baffling.

Studying the pertinent transport phenomena from first principles, the sO_2_ behavior for cases B_0_ and B_2_ (Fig 6) indicates that unless there exists a time delay of the arrival of increased blood flow, relative to CMR_O_2__, no initial dip will the observed. Comparing the no time lag and the 2 s delay scenarios (illustrated in Fig 6) to the BOLD signal duration, it becomes apparent that a time delay in the arrival of increased CBv of about 0.1 s is a precondition for eliciting the initial drop in sO_2_— a correlate of BOLD temporal profile [52–54]. In summary, the overall profile of hemoglobin saturation for cases A, B_0_ to D_0_, as depicted in Fig 6, exhibits excellent correlation with the hemodynamic response of BOLD for the presentation of short duration stimuli.

## Discussion

A spatially resolved computational model of a capillary-tissue system capable of reproducing dynamic transitions in CBv and CMR_O_2__ and mapping the venous fractional hemoglobin saturation, tied to the BOLD hemodynamic response, was presented. We report the first attempt to reproduce hemoglobin saturation, a marker of the BOLD signal, at the single capillary level from first principles utilizing the least number of assumptions possible. Basal and activated states’ sO_2_ profiles and terminal sO_2_ values, for *m* = 2, correlate excellently with physiological hemoglobin saturation profiles. Comparing the temporal behavior between the sO_2_ profile, for *m* = 2, with the BOLD signal response which peaks between 6 to 10 s from stimulus onset [6, 57], the resemblance is evident.

However, caution should be exercised when correlating the modeled hemoglobin saturation with the BOLD MR signal. Notably, the BOLD signal is quantified as percent change in arbitrary units (a.u.) whereas hemoglobin saturation values obtained from the computational model denote fractional values ranging from 0 to 1. Therefore, attributed to the distinct baselines between the BOLD signal, at 0% signal change, and the sO_2_ baseline value of ≈0.5808, no direct comparison between their profiles is advisable. Bridging the gap between the two parameters is the subject of another investigation. Cases *m* = 3 and *m* = 4, despite displaying the same temporal characteristics as *m* = 2, appear to overestimate end sO_2_ levels in relation to other studies [2, 6, 51]. This overestimation may reflect our choice of specific physiological parameter values from a wide range reported in the literature. Higher than physiological erythrocyte oxygenation levels underline the system’s sensitivity to blood flow changes signifying the influence variations in blood hematocrit could have on sO_2_.

The motivation for investigating a wide spectrum of *m* values, ranging from 2 to 4, was twofold. Foremost, it was necessary to benchmark CBv and CMR_O_2__ values, which yielded sO_2_ levels within physiological and temporal limits, against values found in the literature. Second to appraise the responsiveness of the system to basal and activated CMR_O_2__ and CBv changes. Results show that a baseline CMR_O_2__ value of 200 *μ*mol/100g/min and a CBv of 0.6 mm/s generate an sO_2_ level within physiological boundaries. During activation, a 25% increase in CMR_O_2__ to 250 *μ*mol/100g/min and a 50% raise in CBv (to 0.9 mm/s), for *m* = 2 (scenario I), produced sO_2_ levels exhibiting very good temporal correlative agreement with the BOLD hemodynamic response [2, 6, 51].

Although CBF is the prevalent physiological parameter used in *f*MRI investigations, CBv— which also governs blood flow rate— is a more appropriate metric than capillary-level blood flow. Baseline flow velocity values, in perfused discrete capillaries, exhibit remarkable heterogeneity [58]. These velocity variabilities, which can profoundly impact erythrocyte transit time through capillaries, can have a marked effect on oxygen flux reaching the tissue from the exchange vessels. For this reason, we suggest that the capillary transit time and CBv in conjunction with the capillary length are more meaningful parameters to CBF, at these microscopic scales. Given the geometric complexity and random orientation of cerebral capillaries, the accurate conversion of experimentally obtained regional CBF into theoretical CBv remains a challenge.

We argue that at the local microscopic brain level *m*, rather than *n*, is a more objective measure for blood flow studies and coupling and uncoupling comparisons for blood flow and oxygen metabolism at the capillary level. When investigating cerebral perfusion problems of large enough scale (e.g., voxel size >1.5mm^3^), one may utilize cerebral blood flow (CBF, in units of mL/100g/min). However, at the microcirculation level when dealing with discrete blood exchange vessels, cerebral blood velocity (CBv) is a more appropriate quantity. Considering the variability in CBF in the context of capillary recruitment, which is currently a matter of debate, it is hard to extrapolate CBv from CBF. For these reasons we have opted to adopt CBv. Although perfusion measurements, for example using the microsphere technique, permit fine blood flow measurements, currently they are confined to animal models. For human subjects the resolution of blood flow measurements constitutes one of the parameters which could help advance the prevailing understanding pertaining to the blood flow-metabolism relationship.

Another parameter of interest is the initial dip phenomenon. Although not observed in all *f*MRI studies this early ephemeral drop in the MR signal, believed to reflect uncompensated oxygen consumption, may be more closely knit to neuronal activity than to perfusion response. Even though the actual mechanism responsible for the initial dip remains elusive, several physiological changes could be attributed to this occurrence. A reduction in blood flow, an increase in the venous compartment (blood) volume, or an increase in CMR_O_2__ preceding changes in blood flow are among the proposed explanations. Here we investigated the combined effects of increased metabolism and blood velocity. Based on fundamental transport and mass conservation considerations, we postulate that unless there is a time delay in the arrival of increased velocity saturated blood, at the capillary inlet, no initial dip will be observed. The actual values of such time delays, as computed by models of this nature, can guide conjectures regarding the governing mechanisms of neurovascular coupling.

Numerical models such as the one formulated in this study can be used to generate quantitative information pertaining to the time scales of different physiological changes which can offer guidance as to the possible cerebral mechanisms implicated with neurovascular coupling. Moreover, when the diameters of microvessels become comparable to the size of erythrocytes (6–8 *μ*m), the particulate nature of blood in capillaries has a direct influence on the microvascular bed hematocrit [37]. Collectively, we observe that variations in the hematocrit level and the magnitude and duration of CMR_O_2__, and their relative timing, could hinder or promote the appearance of the initial dip. The time window for which the increased CBv supersedes activated CMR_O_2__, after neural activation, coupled with the hematocrit level can alter the dynamics of sO_2_ elucidating also the variability in the BOLD signal duration across different cortical regions and individual subjects.

Model results also provide quantitative evidence that hemoglobin saturation is more responsive to changes in blood velocity than alterations in CMR_O_2__. These findings lend credibility to the hypothesis that increases in CBF is the mechanism which “oversupplies” the microvasculature with oxygenated blood during activation. Extrapolating from this sensitivity of hemoglobin to changes in blood velocity, it is reasonable to infer that a boost in blood velocity can compensate for the absence of capillary recruitment [59]. Note that for resting oxygen metabolism and activated & basal CBv, featured in cases C_0_ and C_2_ & D_0_ and D_2_, as depicted in Fig 6, the rate of change of the hemoglobin saturation curves remained unaltered across *m* = 2, 3 & 4, reflecting thus the importance of the system’s initial oxygen content.

Concluding, tube hematocrit (H_T_) was found to have an appreciable effect on hemoglobin saturation. At the microvascular level hematocrit could be 15% and yet in other cases it could reach normal physiological values of 45%. Modeling results (not presented herein) show that although a H_T_ of 0.31 produces hemoglobin saturation results in accord with physiological measurements, considerable variations from physiological values arise when H_T_ deviates from this value. It might, therefore, be the case that variations in the hematocrit level play a pivotal role in illuminating the inconsistency associated with the manifestation of the initial dip and the BOLD contrast across subjects.
